# Encephalomyeloradiculoneuropathy Revealing a Rare Case of Intravascular Large B-Cell Lymphoma

**DOI:** 10.7759/cureus.12749

**Published:** 2021-01-17

**Authors:** Jehanne Aasfara, Fadila Guessous, Abderahmane Al Bouzidi, Hamid Ouhabi, David Schiff

**Affiliations:** 1 Neurology, Mohammed VI University of Health Sciences (UM6SS) / Cheikh Khalifa International University Hospital, Casablanca, MAR; 2 Biological Sciences, Faculty of Medicine, Mohammed VI University of Health Sciences (UM6SS), Casablanca, MAR; 3 Pathology, Faculty of Medicine, Mohammed VI University of Health Sciences (UM6SS) / Cheikh Khalifa International University Hospital, Casablanca, MAR; 4 Neurology, Faculty of Medicine, Mohammed VI University of Health Sciences (UM6SS) / Cheikh Khalifa International University Hospital, Casablanca, MAR; 5 Neurology, University of Virginia, Charlottesville, USA

**Keywords:** intravascular large b-cell lymphoma, encephalomyelitis, radiculoneuropathy, biopsy

## Abstract

Intravascular large B cell lymphoma (IVLBCL) is a rare form of extranodal non-Hodgkin's lymphoma, usually of B-cell lineage. Several organs are affected, most commonly the skin and the nervous system. We report a case of a 52-year-old man, with no medical history admitted with a five-month history of back pain with lower extremity numbness and tingling evolved to weakness associated with urinary retention, constipation and abdominal pain. Spinal magnetic resonance imaging (MRI) showed a gadolinium-enhancing lesion in the conus medullaris (CM). Electromyography (EMG) and nerve conduction velocity (NCV) test was consistent with demyelinating polyradiculoneuropathy in lower limbs. Slight clinical improvement with corticosteroids was observed. Three months after discharge, he presented a generalized tonic-clonic seizure. Cerebral MRI showed patchy lesions in the subcortical white matter with infiltration of the internal table of the skull with elevated serum lactate dehydrogenase (LDH). Calvarial biopsy revealed an intravascular large B-cell lymphoma. Treatment with cyclophosphamide and high-dose corticosteroids was initiated but the patient developed impaired consciousness and died of respiratory and circulatory failure six weeks after his readmission.

Intravascular large B cell lymphoma should be considered in patients with a rapidly progressive severe encephalomyeloradiculoneuropathy. A biopsy of involved organs including the brain should not be delayed when IVLBCL is suspected, to initiate prompt systemic therapy.

## Introduction

Intravascular large B-cell lymphoma (IVLBCL) is a rare form of extranodal non-Hodgkin's lymphoma characterized by proliferation of malignant lymphocytes in the lumina of small- and medium-sized vessels [1]. Several organs are affected, most commonly the skin and the nervous system. Because of multiple presentations, rarity, and lack of specific radiological and laboratory data, diagnosis is usually difficult and challenging [2].

Encephalomyeloradiculoneuropathy is a rare disorder affecting the central and peripheral nervous systems and likely triggered by an autoimmune or connective tissue disorders, antecedent of vaccination or viral infection [3].

We report an unusual case presenting with encephalomyeloradiculoneuropathy from IVLBCL.

## Case presentation

**Figure 1:**
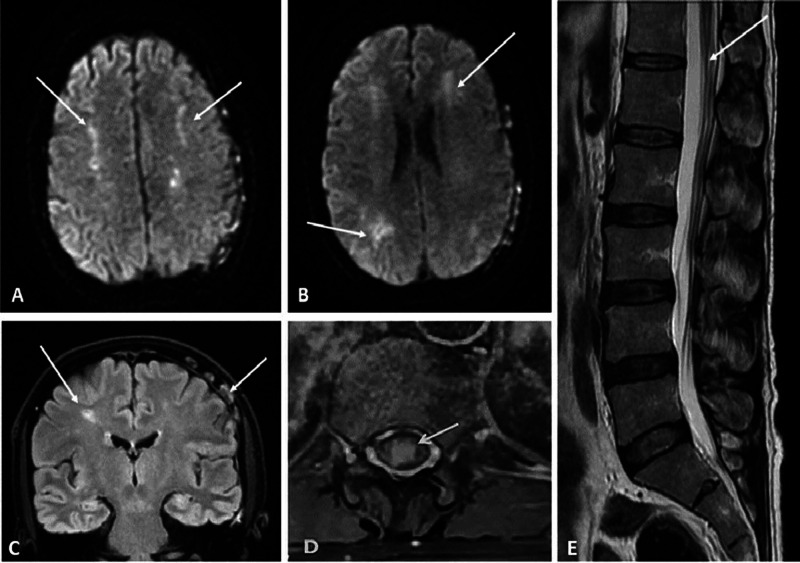
Magnetic resonance imaging of the brain and lumbosacral spine. (A, B) Diffusion-weighted imaging, multiple bilateral asymmetrical predominantly subcortical hyperintense white matter lesions. (C) FLAIR multiple and bilateral hyperintense lesions in the subcortical white matter of the bilateral centrum semiovale and periventricular areas with infiltration of the internal table of the skull. (D) Axial and (E) sagittal T2 weighted imaging signal change in the conus medullaris which enhanced after gadolinium administration.

A previously healthy 52-year-old man was admitted to our neurology department with two months of back pain with lower extremity numbness and tingling that over three months evolved to weakness associated with urinary retention, constipation and abdominal pain. Neurological examination showed flaccid, hypotonic and areflexic paraparesis with 3/5 strength. Abdominal and cremasteric reflexes were absent. There was symmetrical decrease of all sensory modalities below the T10-T11 dermatome. Spinal magnetic resonance imaging (MRI) showed a T2 hyperintense signal of the conus medullaris (CM) which enhanced after gadolinium administration (Figure 1).

Electromyography/nerve conduction velocity (EMG/NCV) was consistent with demyelinating polyradiculoneuropathy in lower limbs (significant reduction of both compound muscle and sensory nerve action potential amplitudes and prolongation of F-wave minimal latencies in lower limb nerves without active denervation of bilateral lower extremity muscles). Brain MRI, visual evoked potentials and lumbar puncture for cerebrospinal fluid analysis (CSF), were performed and were notable only for mildly elevated CSF protein at 0.55 g/L (0.15-0.45); oligoclonal banding was absent.

**Figure 2:**
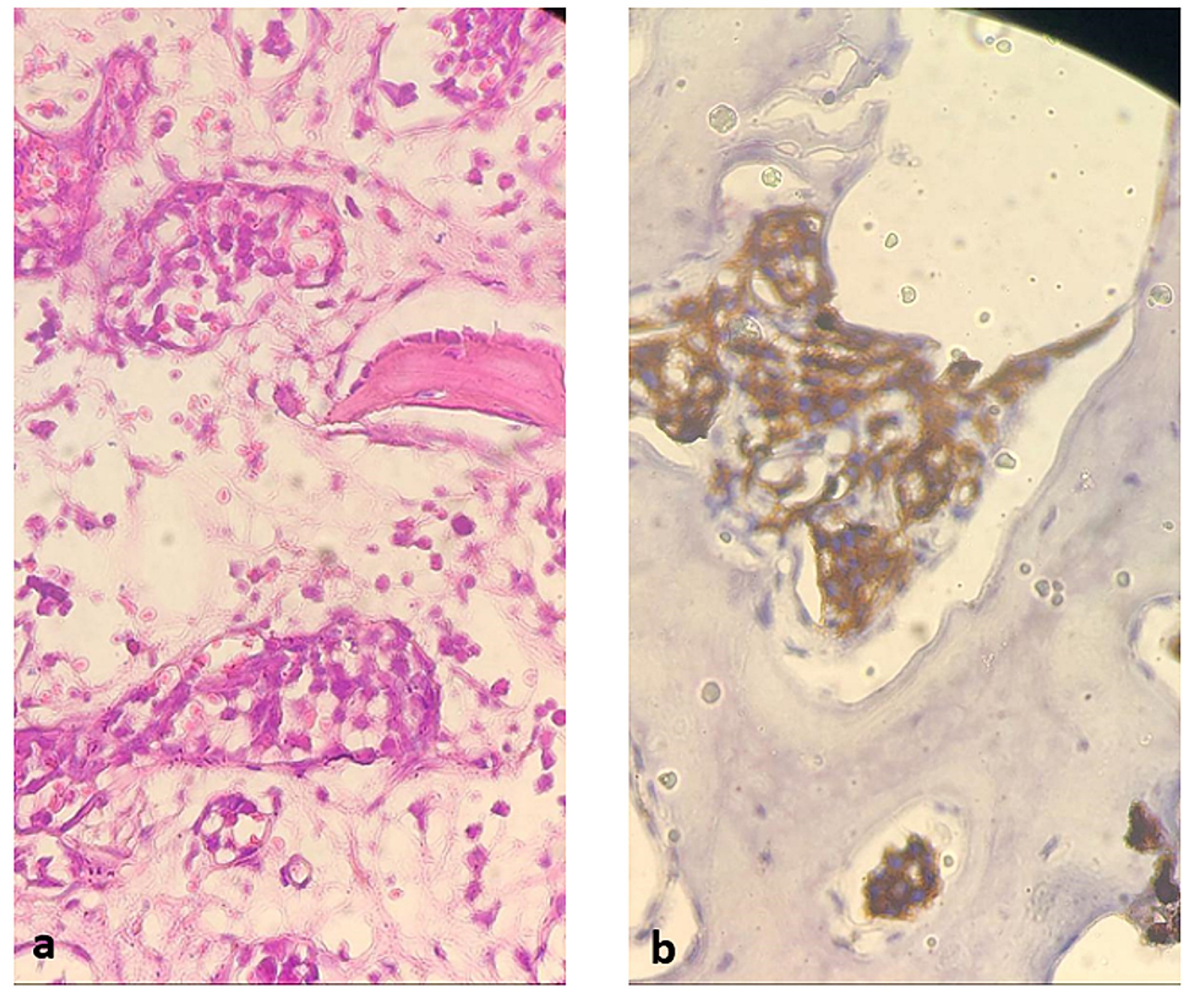
Histological and immunohistochemical findings. (a) Hematoxylin & eosin stained section of temporal muscle shows atrophic fibres along with intravascular large B-cell lymphoma (IVLBCL) (GX40) (b) CD20 immunoreactivity of intravascular large B-cell lymphoma (GX100)

Serum protein immunoelectrophoresis, serum free light chain and LDH levels, angiotensin I-converting enzyme, thyroid-stimulating hormone (TSH), vitamin B12 and folate were normal. All serologies including HIV, varicella zoster virus (VZV), hepatitis B virus (HBV) and hepatitis C virus (HCV), treponema pallidum hemagglutination assay and venereal disease research laboratory (TPHA-VDRL) and Borrelia were negative. Antinuclear, DNA and antineutrophil cytoplasmic antibodies (ANCA) antibodies were all negative. The patient received intravenous methylprednisolone 1 g for three days followed by prednisone (1 mg/kg/d). He regained ambulatory function, although sensory abnormalities and urinary retention persisted. Three months after discharge, he experienced a generalized tonic-clonic seizure treated with lamotrigine. Cerebral MRI showed patchy lesions in the subcortical white matter of the bilateral centrum semiovale and periventricular areas with infiltration of the internal table of the skull (Figure 1). Computed tomography scan of the chest, abdomen and pelvis demonstrated nodular thickening of both kidneys. Serum LDH was now elevated at 1459 U/l (240-480 U/l). Bone marrow biopsy was normal; however, a calvarial biopsy revealed an IVLBCL (Figure 2).

Treatment with cyclophosphamide and high-dose corticosteroids was initiated but the patient developed impaired consciousness and died of respiratory and circulatory failure six weeks after his readmission.

## Discussion

Our case underlines the importance of early diagnosis of IVLBCL, a rare form of extranodal non-Hodgkin's lymphoma, usually of B-cell lineage (85-90% of cases) [1]. This lymphoma is characterized by a proliferation of B-lymphocytes confined to the lumen of small- and medium-sized vessels causing obstruction as well as haemorrhagic lesions in multiple organs, most commonly the skin and the nervous system [2]. The mean age of disease is 70 years (40-80 years) with male predominance. The mortality rate is generally higher than 80% [4,5]. Early diagnosis is challenging because of heterogeneous clinical presentation and lack of specific radiological and laboratory data. Neurological manifestations have been reported in 63% of cases, with subacute encephalopathy, seizure, myelopathy, radiculopathy, and neuropathy were the main associated complications [3].

In our case, encephalomyeloradiculoneuropathy was the initial manifestation. Differential diagnosis included central nervous system (CNS) vasculitis, paraneoplastic and metabolic disorders were considered. The absence of lymphadenopathy and malignant cells in CSF and bone marrow of our patient and most patients with IVLCB, delayed the diagnosis [6,7].

Brain MRI may reveal several patterns of CNS involvement, including nonspecific white matter lesions, widespread enhancement and scattered microinfarcts [8,9]. Biopsy of an involved organ is mandatory to establish the diagnosis. Most frequent sites of biopsy are skin, brain and lung [10].

The management of intravascular lymphoma (IVL) remains controversial and suboptimal in the absence of guidelines or controlled studies. Recent data suggest prognosis may be improving with use of the anti-CD20 monoclonal antibody (rituximab), usually in conjunction with multiagent chemotherapy (cyclophosphamide, doxorubicin, vincristine, prednisone) [11,12].

## Conclusions

Encephalomyeloradiculoneuropathy is an extremely rare presentation of IVLBCL. Neurologists should be aware that multifocal involvement of other organs (lungs, adrenal glands, kidneys, bones, liver) with neurological manifestations should raise a suspicion of lymphoma. A biopsy of involved organs including the brain should not be delayed when IVL is suspected, to initiate prompt systemic therapy.
